# 1,3-Dibenzyl-6-bromo-1*H*-imidazo[4,5-*b*]pyridin-2(3*H*)-one

**DOI:** 10.1107/S1600536810007713

**Published:** 2010-03-06

**Authors:** S. Dahmani, Y. Kandri Rodi, F. Capet, El Mokhtar Essassi, Seik Weng Ng

**Affiliations:** a Laboratoire de Chimie Organique Appliquée, Faculté des Sciences et Techniques, Université Sidi Mohamed Ben Abdallah, Fés, Morocco; b Unité de Catalyse et de Chimie du Solide, Ecole Nationale Supérieure de Chimie de Lille, Lille, France; c Laboratoire de Chimie Organique Hétérocyclique, Pôle de Compétences Pharmacochimie, Université Mohammed V-Agdal, BP 1014 Avenue Ibn Batout, Rabat, Morocco; d Department of Chemistry, University of Malaya, 50603 Kuala Lumpur, Malaysia

## Abstract

The imidazopyridine fused-ring in the title compound, C_20_H_16_BrN_3_O, is planar (r.m.s. deviation = 0.011 Å). The phenyl rings of the benzyl substitutents twist away from the central five-membered ring in opposite directions; the rings are aligned at 61.3 (1) and 71.2 (1)° with respect to this ring.

## Related literature

For the medicinal applications of 1,3-dihydro-imidazo[4,5-*b*]pyridin-2-ones, see: Barraclough *et al.* (1990 ▶); Cundy *et al.* (1997 ▶); Desarro *et al.* (1994 ▶); Liu *et al.* (2008 ▶); Mader *et al.* (2008 ▶); Zaki & Proença (2007 ▶). For the product of the reaction of propargyl bromide with 6-bromo-1,3-dihydro-imidazo[4,5-*b*]pyridin-2-one in DMF at room and high temperatures, see: Dahmani *et al.* (2010*a* ▶,*b* ▶).
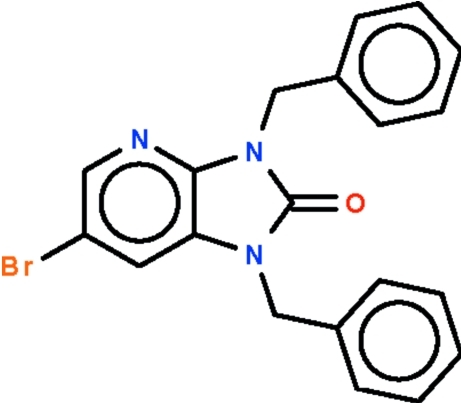


## Experimental

### 

#### Crystal data

C_20_H_16_BrN_3_O
*M* *_r_* = 394.27
Monoclinic, 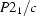

*a* = 9.1627 (1) Å
*b* = 25.5071 (3) Å
*c* = 8.0629 (1) Å
β = 115.571 (1)°
*V* = 1699.84 (3) Å^3^
*Z* = 4
Mo *K*α radiation
μ = 2.43 mm^−1^
*T* = 293 K
0.42 × 0.18 × 0.13 mm

#### Data collection

Bruker X8 APEX2 diffractometer
Absorption correction: multi-scan (*SADABS*; Sheldrick, 1996 ▶) *T*_min_ = 0.428, *T*_max_ = 0.743
38010 measured reflections
3903 independent reflections
2967 reflections with *I* > 2σ(*I*)
*R*_int_ = 0.036

#### Refinement

*R*[*F* ^2^ > 2σ(*F* ^2^)] = 0.041
*wR*(*F* ^2^) = 0.138
*S* = 1.07
3903 reflections
226 parameters
H-atom parameters constrained
Δρ_max_ = 0.84 e Å^−3^
Δρ_min_ = −0.90 e Å^−3^

### 

Data collection: *APEX2* (Bruker, 2008 ▶); cell refinement: *SAINT* (Bruker, 2008 ▶); data reduction: *SAINT*; program(s) used to solve structure: *SHELXS97* (Sheldrick, 2008 ▶); program(s) used to refine structure: *SHELXL97* (Sheldrick, 2008 ▶); molecular graphics: *X-SEED* (Barbour, 2001 ▶); software used to prepare material for publication: *publCIF* (Westrip, 2010 ▶).

## Supplementary Material

Crystal structure: contains datablocks global, I. DOI: 10.1107/S1600536810007713/hg2651sup1.cif

Structure factors: contains datablocks I. DOI: 10.1107/S1600536810007713/hg2651Isup2.hkl

Additional supplementary materials:  crystallographic information; 3D view; checkCIF report

## Figures and Tables

**Fig. 1.**
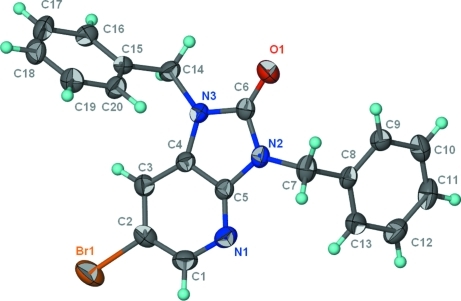
Thermal ellipsoid plot (Barbour, 2001) of C20H16BrN3O at the 50% probability level; hydrogen atoms are drawn as arbitrary radius.

## supplementary crystallographic information

### Comment

Imidazo[4,5-*b*]pyridines are precursors for the synthesis of a variety of medicinal agents as compounds having the imidazo[4,5-*b*]pyridine fused-ring system possess a broad range of pharmacological activities (Barraclough *et al.*, 1990; Cundy *et al.*, 1997; Desarro *et al.*, 1994; Liu *et al.*, 2008; Mader *et al.*, 2008; Zaki & Proença, 2007).

The present study represents the synthesis of the substituted an imidazo[4,5-b]pyridin-2-one derivative by the direct action of benzyl chloride on 6-bromo-1,3-dihydro-imidazo[4,5-*b*]pyridin-2-one in boiling DMF.

The imidazopyridine fused-ring in C_20_H_16_BrN_3_O (Scheme I, Fig. 1) is planar (r.m.s. deviation 0.011 Å). The phenyl rings of the benzyl substitutents twist away from the central five-membered ring in opposite directions; the rings are aligned at 61.3 (1) and 71.2 (1) ° with respect to this ring.

The temperature of the reaction, in the case of propargyl bromide, governs the nature of the product (Dahmani *et al.*, 2010*a*, 2010*b*).

### Experimental

To a mixture of 6-bromo-1,3-dihydro-imidazo[4,5-*b*]pyridin-2-one (1 mmol), potassium carbonate (4 mmol) and benzyltributylammonium chloride (0.1 mmol) in DMF was added benzyl chloride (2.5 mmol). The mixture was stirred for 48 hours. After completion of reaction (monitored by TLC), the inorganic salt was filtered and the solvent was removed under reduced pressure. The residue was purified by column chromatography on silica gel with ethyl acetate/hexane (1/1) as eluent. Colorless crystals were isolated when the solvent was allowed to evaporate.

### Refinement

Carbon-bound H-atoms were placed in calculated positions (C—H 0.93-0.97 Å) and were included in the refinement in the riding model approximation, with *U*(H) set to 1.2*U*(C).

### Crystal data

**Table:**

C_20_H_16_BrN_3_O	*F*(000) = 800
*M**_r_* = 394.27	*D*_x_ = 1.541 Mg m^−^^3^
Monoclinic, *P*2_1_/*c*	Mo *K*α radiation, λ = 0.71073 Å
Hall symbol: -P 2ybc	Cell parameters from 9890 reflections
*a* = 9.1627 (1) Å	θ = 2.5–25.0°
*b* = 25.5071 (3) Å	µ = 2.43 mm^−^^1^
*c* = 8.0629 (1) Å	*T* = 293 K
β = 115.571 (1)°	Prism, colorless
*V* = 1699.84 (3) Å^3^	0.42 × 0.18 × 0.13 mm
*Z* = 4	

### Data collection

**Table:**

Bruker X8 APEX2 diffractometer	3903 independent reflections
Radiation source: fine-focus sealed tube	2967 reflections with *I* > 2σ(*I*)
graphite	*R*_int_ = 0.036
φ and ω scans	θ_max_ = 27.5°, θ_min_ = 1.6°
Absorption correction: multi-scan (*SADABS*; Sheldrick, 1996)	*h* = −11→11
*T*_min_ = 0.428, *T*_max_ = 0.743	*k* = −33→33
38010 measured reflections	*l* = −9→10

### Refinement

**Table:**

Refinement on *F*^2^	Primary atom site location: structure-invariant direct methods
Least-squares matrix: full	Secondary atom site location: difference Fourier map
*R*[*F*^2^ > 2σ(*F*^2^)] = 0.041	Hydrogen site location: inferred from neighbouring sites
*wR*(*F*^2^) = 0.138	H-atom parameters constrained
*S* = 1.07	*w* = 1/[σ^2^(*F*_o_^2^) + (0.0688*P*)^2^ + 1.1691*P*] where *P* = (*F*_o_^2^ + 2*F*_c_^2^)/3
3903 reflections	(Δ/σ)_max_ = 0.001
226 parameters	Δρ_max_ = 0.84 e Å^−^^3^
0 restraints	Δρ_min_ = −0.90 e Å^−^^3^

### Fractional atomic coordinates and isotropic or equivalent isotropic displacement parameters (Å^2^)

**Table:**

	*x*	*y*	*z*	*U*_iso_*/*U*_eq_	
Br1	0.75465 (4)	0.225888 (14)	0.48173 (6)	0.07328 (18)	
O1	0.2072 (3)	0.45942 (8)	0.3332 (3)	0.0570 (5)	
N1	0.3210 (3)	0.28134 (9)	0.4493 (3)	0.0480 (6)	
N2	0.2208 (3)	0.36984 (9)	0.3920 (3)	0.0399 (5)	
N3	0.4318 (3)	0.40833 (8)	0.3767 (3)	0.0398 (5)	
C1	0.4498 (4)	0.25184 (12)	0.4685 (4)	0.0515 (7)	
H1	0.4465	0.2160	0.4879	0.062*	
C2	0.5858 (4)	0.27231 (11)	0.4606 (4)	0.0473 (7)	
C3	0.6008 (3)	0.32553 (11)	0.4321 (4)	0.0428 (6)	
H3	0.6926	0.3396	0.4275	0.051*	
C4	0.4695 (3)	0.35553 (10)	0.4115 (3)	0.0366 (5)	
C5	0.3351 (3)	0.33129 (10)	0.4207 (3)	0.0366 (5)	
C6	0.2777 (3)	0.41774 (10)	0.3635 (4)	0.0401 (6)	
C7	0.0593 (3)	0.36191 (13)	0.3822 (4)	0.0482 (7)	
H7A	0.0226	0.3269	0.3356	0.058*	
H7B	−0.0145	0.3867	0.2952	0.058*	
C8	0.0513 (3)	0.36830 (10)	0.5637 (4)	0.0376 (5)	
C9	0.0052 (3)	0.41597 (11)	0.6102 (4)	0.0448 (6)	
H9	−0.0162	0.4444	0.5310	0.054*	
C10	−0.0090 (4)	0.42129 (13)	0.7723 (4)	0.0530 (7)	
H10	−0.0399	0.4533	0.8023	0.064*	
C11	0.0223 (4)	0.37940 (15)	0.8905 (4)	0.0572 (8)	
H11	0.0105	0.3829	0.9989	0.069*	
C12	0.0711 (4)	0.33231 (14)	0.8480 (5)	0.0581 (8)	
H12	0.0946	0.3042	0.9291	0.070*	
C13	0.0854 (3)	0.32658 (12)	0.6849 (4)	0.0490 (7)	
H13	0.1180	0.2946	0.6565	0.059*	
C14	0.5321 (4)	0.44829 (11)	0.3487 (4)	0.0431 (6)	
H14A	0.6349	0.4498	0.4570	0.052*	
H14B	0.4797	0.4821	0.3362	0.052*	
C15	0.5641 (3)	0.43916 (9)	0.1821 (3)	0.0349 (5)	
C16	0.6900 (3)	0.46603 (11)	0.1695 (4)	0.0437 (6)	
H16	0.7526	0.4889	0.2630	0.052*	
C17	0.7240 (4)	0.45935 (14)	0.0203 (4)	0.0549 (8)	
H17	0.8092	0.4777	0.0137	0.066*	
C18	0.6319 (4)	0.42548 (13)	−0.1197 (4)	0.0533 (7)	
H18	0.6559	0.4204	−0.2194	0.064*	
C19	0.5046 (4)	0.39937 (12)	−0.1105 (4)	0.0498 (7)	
H19	0.4411	0.3770	−0.2055	0.060*	
C20	0.4702 (3)	0.40613 (11)	0.0401 (4)	0.0445 (6)	
H20	0.3836	0.3884	0.0453	0.053*	

### Atomic displacement parameters (Å^2^)

**Table:**

	*U*^11^	*U*^22^	*U*^33^	*U*^12^	*U*^13^	*U*^23^
Br1	0.0600 (2)	0.0554 (2)	0.0961 (3)	0.01808 (15)	0.0259 (2)	0.00089 (17)
O1	0.0630 (13)	0.0486 (12)	0.0681 (14)	0.0147 (10)	0.0364 (11)	0.0062 (10)
N1	0.0528 (14)	0.0437 (13)	0.0526 (14)	−0.0072 (10)	0.0274 (12)	0.0003 (10)
N2	0.0410 (11)	0.0455 (12)	0.0394 (12)	−0.0017 (9)	0.0233 (10)	−0.0020 (9)
N3	0.0454 (12)	0.0371 (11)	0.0455 (12)	−0.0015 (9)	0.0277 (10)	0.0009 (9)
C1	0.0595 (18)	0.0372 (15)	0.0584 (18)	−0.0029 (13)	0.0261 (15)	0.0018 (13)
C2	0.0459 (15)	0.0424 (15)	0.0494 (16)	0.0071 (12)	0.0166 (13)	−0.0007 (12)
C3	0.0398 (13)	0.0436 (14)	0.0478 (15)	0.0001 (11)	0.0214 (12)	−0.0026 (12)
C4	0.0424 (13)	0.0371 (12)	0.0336 (12)	−0.0029 (10)	0.0194 (11)	−0.0028 (10)
C5	0.0392 (13)	0.0400 (13)	0.0336 (12)	−0.0026 (10)	0.0186 (10)	−0.0031 (10)
C6	0.0482 (15)	0.0438 (14)	0.0346 (13)	0.0014 (12)	0.0239 (11)	−0.0020 (11)
C7	0.0359 (13)	0.0663 (19)	0.0426 (15)	−0.0056 (12)	0.0172 (12)	−0.0081 (13)
C8	0.0291 (11)	0.0463 (14)	0.0404 (13)	−0.0053 (10)	0.0177 (10)	−0.0025 (11)
C9	0.0402 (14)	0.0445 (15)	0.0506 (16)	0.0020 (11)	0.0204 (12)	0.0050 (12)
C10	0.0482 (16)	0.0594 (18)	0.0570 (18)	0.0009 (14)	0.0279 (14)	−0.0135 (15)
C11	0.0468 (16)	0.090 (2)	0.0415 (16)	−0.0001 (16)	0.0251 (13)	−0.0038 (16)
C12	0.0531 (18)	0.069 (2)	0.0566 (19)	0.0009 (15)	0.0276 (15)	0.0177 (16)
C13	0.0460 (15)	0.0441 (15)	0.0637 (18)	0.0007 (12)	0.0302 (14)	0.0014 (13)
C14	0.0544 (16)	0.0379 (13)	0.0447 (15)	−0.0103 (12)	0.0287 (13)	−0.0057 (11)
C15	0.0382 (12)	0.0309 (12)	0.0383 (13)	0.0023 (10)	0.0190 (11)	0.0031 (10)
C16	0.0412 (14)	0.0442 (15)	0.0437 (15)	−0.0059 (11)	0.0166 (12)	0.0001 (11)
C17	0.0486 (17)	0.066 (2)	0.0605 (19)	−0.0051 (14)	0.0338 (16)	0.0068 (15)
C18	0.0600 (18)	0.0642 (19)	0.0467 (16)	0.0081 (15)	0.0333 (15)	0.0053 (14)
C19	0.0571 (17)	0.0519 (16)	0.0401 (15)	−0.0015 (13)	0.0206 (13)	−0.0074 (12)
C20	0.0460 (15)	0.0461 (15)	0.0463 (15)	−0.0085 (12)	0.0245 (13)	−0.0048 (12)

### Geometric parameters (Å, °)

**Table:**

Br1—C2	1.897 (3)	C9—H9	0.9300
O1—C6	1.213 (3)	C10—C11	1.377 (5)
N1—C5	1.311 (3)	C10—H10	0.9300
N1—C1	1.351 (4)	C11—C12	1.376 (5)
N2—C5	1.382 (3)	C11—H11	0.9300
N2—C6	1.386 (3)	C12—C13	1.384 (5)
N2—C7	1.462 (3)	C12—H12	0.9300
N3—C4	1.389 (3)	C13—H13	0.9300
N3—C6	1.391 (3)	C14—C15	1.512 (4)
N3—C14	1.453 (3)	C14—H14A	0.9700
C1—C2	1.377 (4)	C14—H14B	0.9700
C1—H1	0.9300	C15—C20	1.382 (4)
C2—C3	1.393 (4)	C15—C16	1.383 (4)
C3—C4	1.374 (4)	C16—C17	1.377 (4)
C3—H3	0.9300	C16—H16	0.9300
C4—C5	1.408 (4)	C17—C18	1.382 (5)
C7—C8	1.505 (4)	C17—H17	0.9300
C7—H7A	0.9700	C18—C19	1.372 (5)
C7—H7B	0.9700	C18—H18	0.9300
C8—C13	1.386 (4)	C19—C20	1.391 (4)
C8—C9	1.390 (4)	C19—H19	0.9300
C9—C10	1.376 (4)	C20—H20	0.9300
			
C5—N1—C1	114.4 (2)	C9—C10—C11	120.3 (3)
C5—N2—C6	110.0 (2)	C9—C10—H10	119.9
C5—N2—C7	126.0 (2)	C11—C10—H10	119.9
C6—N2—C7	123.9 (2)	C12—C11—C10	119.9 (3)
C4—N3—C6	109.8 (2)	C12—C11—H11	120.1
C4—N3—C14	126.4 (2)	C10—C11—H11	120.1
C6—N3—C14	123.8 (2)	C11—C12—C13	120.2 (3)
N1—C1—C2	123.1 (3)	C11—C12—H12	119.9
N1—C1—H1	118.4	C13—C12—H12	119.9
C2—C1—H1	118.4	C12—C13—C8	120.2 (3)
C1—C2—C3	122.2 (3)	C12—C13—H13	119.9
C1—C2—Br1	118.6 (2)	C8—C13—H13	119.9
C3—C2—Br1	119.2 (2)	N3—C14—C15	114.1 (2)
C4—C3—C2	114.8 (3)	N3—C14—H14A	108.7
C4—C3—H3	122.6	C15—C14—H14A	108.7
C2—C3—H3	122.6	N3—C14—H14B	108.7
C3—C4—N3	133.9 (2)	C15—C14—H14B	108.7
C3—C4—C5	119.3 (2)	H14A—C14—H14B	107.6
N3—C4—C5	106.9 (2)	C20—C15—C16	118.8 (2)
N1—C5—N2	126.6 (2)	C20—C15—C14	122.7 (2)
N1—C5—C4	126.2 (3)	C16—C15—C14	118.5 (2)
N2—C5—C4	107.2 (2)	C17—C16—C15	120.9 (3)
O1—C6—N2	126.9 (3)	C17—C16—H16	119.6
O1—C6—N3	127.0 (3)	C15—C16—H16	119.6
N2—C6—N3	106.1 (2)	C16—C17—C18	120.1 (3)
N2—C7—C8	113.9 (2)	C16—C17—H17	120.0
N2—C7—H7A	108.8	C18—C17—H17	120.0
C8—C7—H7A	108.8	C19—C18—C17	119.6 (3)
N2—C7—H7B	108.8	C19—C18—H18	120.2
C8—C7—H7B	108.8	C17—C18—H18	120.2
H7A—C7—H7B	107.7	C18—C19—C20	120.3 (3)
C13—C8—C9	118.9 (2)	C18—C19—H19	119.8
C13—C8—C7	120.7 (3)	C20—C19—H19	119.8
C9—C8—C7	120.3 (3)	C15—C20—C19	120.3 (3)
C10—C9—C8	120.4 (3)	C15—C20—H20	119.9
C10—C9—H9	119.8	C19—C20—H20	119.9
C8—C9—H9	119.8		
			
C5—N1—C1—C2	0.6 (4)	C4—N3—C6—N2	0.4 (3)
N1—C1—C2—C3	0.0 (5)	C14—N3—C6—N2	177.7 (2)
N1—C1—C2—Br1	−178.0 (2)	C5—N2—C7—C8	92.2 (3)
C1—C2—C3—C4	−0.4 (4)	C6—N2—C7—C8	−91.5 (3)
Br1—C2—C3—C4	177.6 (2)	N2—C7—C8—C13	−86.0 (3)
C2—C3—C4—N3	−178.0 (3)	N2—C7—C8—C9	95.7 (3)
C2—C3—C4—C5	0.1 (4)	C13—C8—C9—C10	−1.1 (4)
C6—N3—C4—C3	178.2 (3)	C7—C8—C9—C10	177.2 (3)
C14—N3—C4—C3	0.9 (5)	C8—C9—C10—C11	0.0 (4)
C6—N3—C4—C5	−0.1 (3)	C9—C10—C11—C12	1.3 (5)
C14—N3—C4—C5	−177.4 (2)	C10—C11—C12—C13	−1.4 (5)
C1—N1—C5—N2	178.3 (3)	C11—C12—C13—C8	0.3 (5)
C1—N1—C5—C4	−1.0 (4)	C9—C8—C13—C12	1.0 (4)
C6—N2—C5—N1	−178.9 (3)	C7—C8—C13—C12	−177.3 (3)
C7—N2—C5—N1	−2.1 (4)	C4—N3—C14—C15	62.6 (4)
C6—N2—C5—C4	0.5 (3)	C6—N3—C14—C15	−114.3 (3)
C7—N2—C5—C4	177.3 (2)	N3—C14—C15—C20	18.6 (4)
C3—C4—C5—N1	0.6 (4)	N3—C14—C15—C16	−163.1 (2)
N3—C4—C5—N1	179.2 (3)	C20—C15—C16—C17	−1.3 (4)
C3—C4—C5—N2	−178.8 (2)	C14—C15—C16—C17	−179.7 (3)
N3—C4—C5—N2	−0.2 (3)	C15—C16—C17—C18	0.1 (5)
C5—N2—C6—O1	179.4 (3)	C16—C17—C18—C19	1.2 (5)
C7—N2—C6—O1	2.6 (4)	C17—C18—C19—C20	−1.2 (5)
C5—N2—C6—N3	−0.5 (3)	C16—C15—C20—C19	1.4 (4)
C7—N2—C6—N3	−177.4 (2)	C14—C15—C20—C19	179.7 (3)
C4—N3—C6—O1	−179.6 (3)	C18—C19—C20—C15	−0.1 (5)
C14—N3—C6—O1	−2.2 (4)		
